# Chronic Carcinogenicity Study of Gasoline Vapor Condensate (GVC) and GVC Containing Methyl Tertiary-Butyl Ether in F344 Rats

**DOI:** 10.1080/15287394.2011.538837

**Published:** 2011-03-22

**Authors:** Janet M. Benson, Andrew P. Gigliotti, Thomas H. March, Edward B. Barr, Brad M. Tibbetts, Betty J. Skipper, Charles R. Clark, Lorraine Twerdok

**Affiliations:** ^a^ Lovelace Respiratory Research Institute, Albuquerque, New Mexico; ^b^ Department of Family and Community Medicine, University of New Mexico, Albuquerque, New Mexico; ^c^ ConocoPhillips, Industrial Hygiene and Toxicology, Bartlesville, Oklahoma; ^d^ Toxicology Consulting Services, Silver Spring, Maryland, USA

## Abstract

Chronic inhalation studies were conducted to compare the toxicity and potential carcinogenicity of evaporative emissions from unleaded gasoline (GVC) and gasoline containing the oxygenate methyl tertiary-butyl ether (MTBE; GMVC). The test materials were manufactured to mimic vapors people would be exposed to during refueling at gas stations. Fifty F344 rats per gender per exposure level per test article were exposed 6 h/d, 5 d/wk for 104 wk in whole body chambers. Target total vapor concentrations were 0, 2, 10, or 20 g/m^3^ for the control, low-, mid-, and high-level exposures, respectively. Endpoints included survival, body weights, clinical observations, organs weights, and histopathology. GVC and GMVC exerted no marked effects on survival or clinical observations and few effects on organ weights. Terminal body weights were reduced in all mid- and high-level GVC groups and high-level GMVC groups. The major proliferative lesions attributable to gasoline exposure with or without MTBE were renal tubule adenomas and carcinomas in male rats. GMV exposure led to elevated testicular mesothelioma incidence and an increased trend for thyroid carcinomas in males. GVMC inhalation caused an increased trend for testicular tumors with exposure concentration. Mid- and high-level exposures of GVC and GMVC led to elevated incidences of nasal respiratory epithelial degeneration. Overall, in these chronic studies conducted under identical conditions, the health effects in F344 rats following 2 yr of GVC or GMVC exposure were comparable in the production of renal adenomas and carcinomas in male rats and similar in other endpoints.

The 1990 Clean Air Act (CAA) amendments mandated the use of oxygenates in motor gasoline. The CAA also requires manufacturers of fuels and fuel additives to provide data to the U.S. Environmental Protection Agency (EPA) regarding the potential health effects of their products (40 CFR, Subpart F; current as of December 15, 2010). Chemical and biological characterization of the evaporative emissions of 1990 industry average gasoline and oxygenated gasoline were included in these requirements in 1997 (40 CFR 79.58(c); current as of December 15, 2010). Toxicology studies to characterize the health hazards of evaporative emissions were conducted by a consortium of fuel and fuel additive manufacturers and were coordinated by the American Petroleum Institute. The results of chronic inhalation studies of vapor condensates generated from gasoline (gasoline vapor condensate; GVC) and gasoline containing 15% (by volume) methyl tertiary-butyl ether (MTBE; gasoline + MTBE vapor condensate; GMVC) are presented. These studies were conducted in conformance with Good Laboratory Practice (GLP) Guidelines (40 CFR 79.60; current as of December 15, 2010).

Methyl tertiary-butyl ether (MTBE) was the most commonly used oxygenate in the 1990s, but its use was dramatically reduced in the subsequent decade. Inhalation is the most common route of exposure to workers and the general public. Worker exposure may occur during manufacture and transport of MTBE-containing fuels. Inhalation of vapors while refueling automobiles is the predominant route of exposure for the general public (HEI, 2001; Ahmed, 2001), with concentrations measured near the car during approximately 3 min of refueling with gasoline containing MTBE ranging from 0.1 to 38 ppm (0.36 to 138 mg/m^3^; HEI, 2001). Concentrations of hydrocarbon vapor encountered during refueling have also been measured. In one study, total hydrocarbon vapor from unleaded gasoline measured during 2 min of self-service fill-up was 10–100 ppm (Clayton Environmental Consultants, 1993). In an earlier study, conducted during fueling test cars with unleaded gasoline, no vapor exposure was detected during up to 22 min of refueling (4 cars fueled with a total of 17 gallons of gasoline). A 27-min exposure (6 cars fueled with 25 gallons of fuel) resulted in 4–8 ppm hydrocarbon exposure (12.7–24.3 mg/m^3^; Halder et al., 1986).

Subchronic and chronic inhalation studies of MTBE were conducted in rodents (Ahmed, 2001). Of direct relevance to the studies reported here is the oncogenicity study of MTBE conducted in CD-1 mice and F344 rats (Bird et al., 1997). In that study, rats and mice were exposed 6 h/d, 5 d/wk for 18 mo (mice) or 24 mo (rats) to 400, 3000, or 8000 ppm MTBE vapor (approximately 1.4–29 g MTBE/m^3^). The highest concentration tested was approximately 50% of the lower explosive limit. The lowest concentration was approximately 10-fold higher than that measured at gas stations during refueling (HEI, 1996).

Male rats exposed to 3000 and 8000 ppm experienced excess mortality due to severe progressive nephrosis, which was not observed among females. At terminal sacrifice, chronic progressive nephropathy (CPN) was observed in all MTBE-exposed males and in females exposed to 3000 and 8000 ppm. Renal tubular-cell tumors and interstitial-cell adenomas of the testes were significantly increased in male rats exposed to 3000 and 8000 ppm. The testicular lesions are common among aged rats, and the significant increase seen among high dose males was attributed to an unexpected low incidence in control males. The no-observed-effect level (NOEL) for cancer in rats was 400 ppm.

Previous studies evaluated the toxicity of chronically inhaled wholly vaporized gasoline (MacFarland et al., 1984). In this published study, F344 rats and B6C3F1 mice were exposed 6 h/d, 5 d/wk for 24 to 27 mo to 67, 292 or 2056 ppm (approximately 0.2–6.3 g/m^3^) wholly vaporized unleaded gasoline. As in the chronic MTBE inhalation study, the most significant findings were observed in male rat kidneys. Male rats sacrificed during the first 6 mo exhibited what was described at the time as a “progressive renal tubular disease.” At the conclusion of the study, renal carcinomas, sarcomas, or adenomas were detected in male rats at all dose levels and in one female rat at the intermediate dose level.

The objective of the current study was to evaluate the potential adverse health effects of gasoline vapor in F344 rats and determine whether the addition of MTBE to the test substance changed the response.

## METHODS

### Test Materials

Gasoline vapor condensate (GVC, lots API 99-01 and API 02-08) and gasoline MTBE vapor condensate (GMVC, lot API 00-02) were prepared and supplied in 420-lb and 20-lb gas cylinders by Chevron Research and Technology Center (CRTC, Richmond, CA).

The test materials were fabricated to mimic vapors people would be exposed to during refueling of their vehicle. The starting gasoline for the generation of both vapor condensate samples was certified to meet the 1990 industry average gasoline properties in 40 CFR 79.55. To generate GMVC, MTBE was splash blended into the starting gasoline to achieve 2.7% oxygen by weight (14.9% MTBE by volume) and carried through the vapor generation and collection process described by Daughtrey et al. (2004). Briefly, vapor condensate was generated by a single-step distillation from a 1000-gal Pfaudler glass-lined kettle wherein approximately 15 to 23% of the starting material was slowly vaporized, separated, condensed by chilling, and recovered as test sample. The liquid temperature during collection was approximately 66°C, which resulted in a vapor temperature of approximately 54°C.

Original characterization of the test substances was performed by ExxonMobil Biomedical Sciences, Inc. (EMBSI, 2009). The compositions of the major components of the condensate samples are compared in Table 1. While there were some differences in the relative concentrations of various alkanes and isoalkane components in the two lots of GVC used, these differences are not believed to have influenced the outcome of the study. The analysis showed that the relative concentration of MTBE in the GMVC was 21.3%.

**TABLE 1.** Analytical Comparison of Test Materials

Compound	GVC lot API 02-08 (area percent)^*a*^	GVC lot API 99-01 (area percent)^*b*^	GMVC lot API 00-02 (area percent)
Isobutane	2.1	3.6	2.2
*n*-Butane	19.9	15.2	11.1
Isopentane	32.0	35.1	31.0
*n*-Pentane	5.4	13.2	9.1
*Trans*-2-pentene	2.4	2.5	2.0
2-Methyl-2-butene	3.3	3.8	2.9
MTBE	–	–	21.3
2,3-Dimethylbutane	6.2	1.6	0.9
2-Methylpentane	9.6	6.3	4.5
3-Methylpentane	6.8	3.6	2.6
*n*-Hexane	1.2	3.0	2.1
Methylcyclopentane	0.7	1.5	1.1
2,4-Dimethylpentane	0.6	1.0	0.9
Benzene	1.9	2.1	1.5
2-Methylhexane	1.6	1.1	1.0
2,3-Dimethylpentane	1.0	1.1	1.0
3-Methylhexane	2.2	1.3	1.1
Isooctane	0.7	1.3	1.2
Toluene	2.6	3.0	2.5
^*a*^Used from May 27, 2003–August 15, 2003.			
^*b*^Used from August 13, 2001–May 23, 2003.			

Twenty-pound cylinders and some 420-lb cylinders were stored at ambient temperature in a dedicated storage building. The remaining 420-lb cylinders were stored in an outside, controlled area at ambient temperature. The test substance was transferred, as needed, from the 420-lb to the 20-lb cylinders. Before dispensing the test article from each 420-lb tank, a sample was removed from the tank and analyzed by gas chromatography (GC) with flame ionization detection (FID) using a Shimadzu model GC-17A/FID (Shimadzu Scientific Instruments, Columbia, MD). The gas chromatographic profile of the 18 major peaks (retention time and relative peak area) was compared with that originally determined for the GVC and GMVC by ExxonMobil Biomedical Sciences, Inc. (EMBSI, 2009), to ensure the stability of the test mixtures throughout the study.

### Animals

Four hundred and forty CDF(F344)CrlBR rats (5–6 wk old, weighing approximately 100 g when received) were purchased from Charles River Laboratories (Raleigh, NC) for each study. All animals were quarantined and acclimated to whole-body inhalation chambers for at least 14 d. Healthy animals were randomly assigned by weight to the core exposure groups (400 total; 50 rats/gender/exposure level/test material). Following randomization, the rats assigned to study were identified by tail tattoo. Five unassigned male and female rats were sacrificed for each study before exposures began to evaluate their health status as an indicator of the health status of the population on study. Five male and five female rats for each study were assigned as sentinels and housed in the respective control chamber. Sentinels were screened for health status prior to the beginning of the study and at 6-mo intervals. Blood was collected via retroorbital puncture while the animals were under halothane anesthesia. Serum was harvested and submitted to BioReliance, Rockville, MD, for analysis of antibodies against common rodent pathogens.

### Environmental Conditions

The rats were housed 24 h/d during quarantine and exposure in Hazleton 2000 whole-body inhalation chambers. Initially, all rats were housed separately in compartments 9.7 cm wide by 27.9 cm long by 20.3 cm high within stainless-steel baskets. When the male rats reached 400 g, they were transferred to baskets with 14.5- by 27.9- by 20.3-cm compartments. Each chamber contained six baskets.

The chambers were held at negative pressure with respect to the exposure room, and the chamber flow rates were maintained at 12 to 15 air changes per hour (400–500 L/min [lpm]). Chamber temperatures were maintained at 20 to 24°C. Temperature, relative humidity, and chamber airflow rates were continuously monitored, 24 h/d. Values for the three parameters were recorded at 30-min intervals. Oxygen concentration in the chambers was maintained at 19%. A 12-h light/dark cycle was maintained with lights on at 0600. Light levels in the exposure room and noise levels in the chambers were determined periodically.

### Diet and Drinking Water

Unlimited municipal tap water was available at all times. Rats were fed Teklad certified rodent diet (8728C; Harlan Teklad, Madison, WI). Food was available at all times except during the daily exposure period.

### Experimental Design

Fifty rats per gender per exposure level per test article were exposed 6 h/d (plus 14 min_,_ the time for the vapor concentration to reach 90% of equilibrium), 5 d/wk for 104 wk (518 exposure days for BGMV and 520 exposure days for GMVC) in Hazleton 2000 whole-body chambers. Target total hydrocarbon concentrations were 0, 2, 10, or 20 g/m^3^ for the control, low-, mid-, and high-level exposures respectively. For vapor containing MTBE, the corresponding MTBE concentrations were approximately 0.4, 2, and 4 g/m^3^.

### Inhalation Exposure System

The daily supply of test article for each exposure chamber was contained in 20-lb gas storage cylinders. Exposure atmospheres were generated by controlling the flow of pressurized test article through a rotameter, into a heated stainless-steel transfer line where the test article was completely vaporized. Chamber concentrations were controlled by adjusting the flow rates of the test article and dilution air rate. Chamber exhaust was carried to an oxidizer on the roof of the exposure facility where it was burned.

### Vapor Generation and Characterization

Vapor concentrations in the exposure chambers were continuously monitored using Miran 1A infrared analyzers (Foxboro Wilks, Foxboro, CT). The high-, mid-, and low-level exposure chambers were each monitored with their own analyzer. A fourth analyzer was devoted to monitoring the control chamber, the room air, and the hood enclosing the 20-lb tank of test substance. The Miran analyzers were operated at a wavelength of 10.4 μm. The path length for the instrument monitoring the low chamber was 15.7 m, while the path length for the instruments monitoring the mid- and high-level chambers was 6.75 m. The Miran 1A analyzers dedicated to the mid- and high-level chambers were calibrated using test article over a range of 6–35 g/m^3^. The Miran 1A analyzers for the control chamber and the low-level chamber were calibrated over a concentration range of 1–7 g/m^3^. The exposure atmosphere in the animals' breathing zone of the high level chambers was also examined for the presence of aerosol particles using a TSI scanning mobility particle sizer (TSI Industries, Shoreview, MN). No aerosol was detected.

#### 

##### Qualitative assessment of exposure atmospheres

The qualitative composition of the exposure atmosphere in each chamber was determined weekly by gas chromatography using a Shimadzu Model GC-17A/FID. The percent peak area of each of 18 major components was determined and recorded weekly.

##### Determination of nominal concentration

Daily nominal or “anticipated” usage was calculated by multiplying the average test article concentration in each chamber (low, mid, high; g/m^3^) by the total flow through each respective chamber ([L/min × min]/1000 m^3^) and then summing the values for all three chambers. This value was compared to the actual test article usage determined by taking the difference between the weight of the 20-lb cylinder before and after each exposure.

### In-Life Observations

All animals were individually weighed using the Path-Tox data acquisition system (Version 4.2.2., Xybion, Cedar Knolls, NJ) on study day −7 (to randomly assign rats to groups by weight), d −1, weekly for 13 wk, and then every 4 wk thereafter. Thorough clinical examinations were made at randomization, on d −1, and weekly thereafter.

### Postexposure Endpoints

#### 

##### Gross necropsy

A complete gross examination was performed on all animals at final sacrifice and on those animals that died naturally or were sacrificed in a moribund condition. Sacrifices of rats surviving 518 or 520 d of exposure occurred during the week following the last exposure day for each gender. Animals were randomly assigned to a sacrifice day.

##### Necropsy, lung harvest, and tissue processing

All study animals received a complete necropsy. Animals were euthanized with an overdose of intraperitoneally injected barbiturate anesthetic (Euthasol, Virbac AH, Inc., Fort Worth, TX). Body weights and fresh organ weights were collected on lung, liver, kidneys, adrenals, testes, epididymides, ovaries, uteri, spleen, brain, and heart of final sacrifice and moribund sacrifice animals. Animals found dead received a complete necropsy with tissue collection.

Lungs were gently instilled via the trachea with 10% neutral buffered formalin (NBF) to approximate normal volume. Organs and tissues were immersion fixed in 10% NBF for subsequent histopathologic examination. Tissues were trimmed, processed routinely, paraffin embedded, sectioned at 5 μm, and stained with hematoxylin and eosin for histopathologic examination.

##### Histopathology

All collected tissues and lesions were examined histologically in control (0 g/m^3^) animals, high-level (20 g/m^3^) animals, and dead or moribund animals of all groups. Respiratory tissues (lung, larynx, trachea, and nasal turbinate sections at four levels), potential target tissues (testes, kidneys of males), and gross lesions from nontarget tissues were examined histologically from final sacrifice low-level (2 g/m^3^) and mid-level (10 g/m^3^) animals. Nomenclature of proliferative lesions was based on the international harmonized nomenclature recommended by the Rat Nomenclature Reconciliation Subcommittee of the Society of Toxicologic Pathologists (see http://www.toxpath.org; Standardized Rat Nomenclature). Nomenclature for other lesions was routine, widely understood usage (Boorman et al., 1990a).

### Statistical Analysis

#### 

##### Body and organ weights

Group mean body weight, organ weight, percent organ-to-body weight, and percent organ-to-brain weight data were tested for statistical significance using Path-Tox software. After testing for an overall trend among test groups by an analysis of variance, Bartlett's test was used to establish the homogeneity of the data. If the data were homogeneous, differences between the exposed and control groups were evaluated using a modified Dunnett's test. If data were nonhomogeneous, group differences were assessed using a modified *t*-test. Significance levels were set at *p* ≤ .05.

##### Survival analysis

The probability of survival was estimated by the Kaplan–Meier product-limit method using PROC LIFETEST in SAS Version 8.2 (SAS Institute, Cary, NC). Mean numbers of survival days and time to 25% mortality were estimated for each exposure group by the PROC LIFETEST program. Log-rank tests were used to test the hypothesis that there are differences among the four groups for each gender. The significance level was set at *p* = .05. All reported *p* values for the survival analysis are two-sided.

##### Histopathology

The incidences of all neoplastic and nonneoplastic lesions are given as the ratio of the number of affected animals to the number of animals with the site examined microscopically. Three statistical evaluations were performed on the histopathology lesion incidence data: (1) the Cochran–Armitage test, which tests whether the incidence of lesions shows a trend across exposure groups; (2) a logistic regression test that takes death date into account when assessing the presence of an exposure-dependent trend; and (3) Fisher's exact test, which compares incidences among the four exposure groups. The two-sided significance level was set at *p* = .05. If a significant difference was detected by the Fisher's exact test, six possible pairwise comparisons were calculated. Using the Bonferroni correction for pairwise comparisons, each pair-wise comparison would be considered significant if *p* < .008.

Fisher's exact test and the Cochran–Armitage test do not use survival information and are appropriate in situations where survival is similar among exposure groups, as is the case for this study. The exact test tests the null hypothesis of equality of prevalences across exposure groups against the alternate hypothesis that the prevalences are not equal, while the Cochran–Armitage analysis tests the null hypothesis of equality across exposures against the alternate hypothesis of a monotonic increasing or decreasing trend. Additionally, differences between groups with regard to both the severity and incidence of nonproliferative lesions were analyzed by the Kolmogorov–Smirnov two-sample, one-tailed test as performed by the Path-Tox system. The significance level was set at *p* = .05.

## RESULTS

### Vapor Atmosphere

#### 

##### Chamber concentrations and nominal usage

The overall study means of the daily vapor concentrations achieved for 2, 10, and 20 g/m^3^ for GVC and GMVC are provided in Table 2. In both studies, the overall achieved means were within 2% of target for each exposure concentration. Concentrations of vapor in the control chamber were below the lowest concentration on the standard curve used to calibrate the control chamber Miran 1A analyzer (1 g test article/m^3^). The overall average of the daily percent nominal usage is 98 ± 6% for GVC and 99 ± 4% for GMVC, indicating reliable agreement between anticipated and actual test article usage.

**TABLE 2.** Summary of Test Article Vapor Concentrations

	GVC		GMVC	
Target (g/m^3^)	Achieved concentrations	Percent of target	Achieved concentrations	Percent of target
0	<1^*a*^	NA	NA^*b*^	NA
2	2.02 ± 0.07	101	2.02 ± 0.07	101
10	10.1 ± 0.34	101	10.0 ± 0.37	100
20	20.3 ± 0.74	101	20.3 ± 0.74	101
*Note.* Results are the mean ± SD of vapor concentrations obtained in 518 exposure days (combined days for males and females) for GVC and 520 exposure days for GMCV.				
^*a*^Concentrations of “zero” were obtained on all but 13 exposure days. On these 13 days, the readings were below the lowest concentration on the standard curve used to calibrate the control chamber Miran 1A (1 g GVC/m^3^).				
^*b*^Values were below 1 g GMVC/m^3^, lowest concentration on the standard curve used to calibrate the control chamber Miran 1A.				

### Survival Analysis

Figure 1 shows the survival curves for male and female animals in the GVC (Figure 1, a and b) and GMVC (Figure 1, c and d) studies, respectively. The survival differences among the GVC groups are not statistically significant for male or female animals. The survival differences among the GMVC groups are not statistically significant for male or female animals. Table 3 shows means, standard errors, and day of 25% mortality for male and female animals under each experimental condition. The results corroborate that chronic GVC or GMVC inhalation did not shorten the lifespan of the exposed rats compared to the controls.

**Figure:**
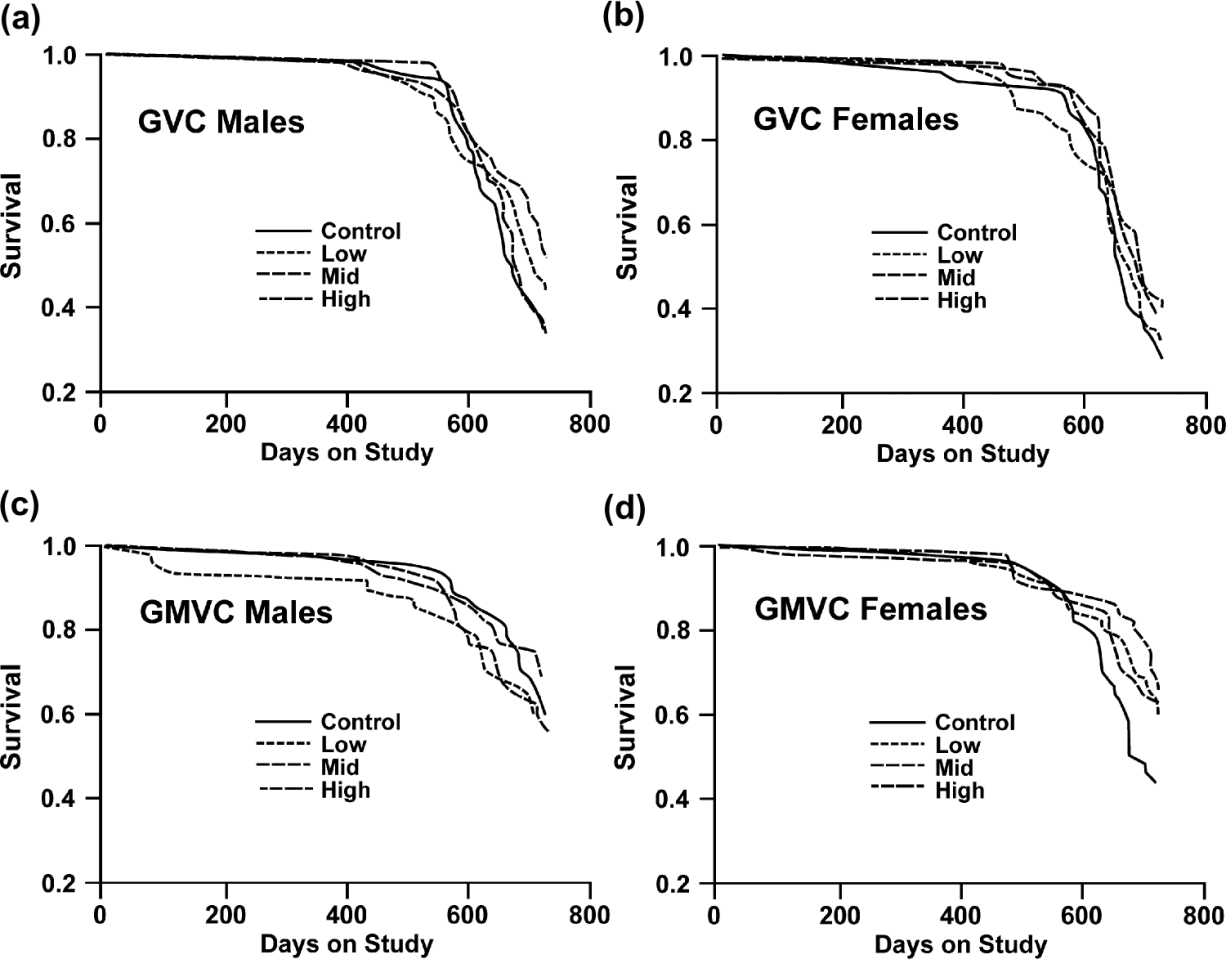
**FIGURE 1.** Survival curves: (a) Survival of GVC male animals. (b) Survival of GVC female animals. (c) Survival of GMVC male animals. (d) Survival of GMVC female animals.

**TABLE 3.** Mean Survival Days and Day of 25% Mortality for Male and Female Rats

	Male		Female	
Group	Mean days of survival (SE)	Estimated day of 25% mortality	Mean Days of survival (SE)	Estimated day of 25% mortality
GVC				
Control	660.5 (10.2)	613	686.8 (11.4)	680
Low	666.5 (12.1)	607	642.9 (24.1)	619
Mid	679.9 (11.6)	642	674.8 (12.2)	640
High	669.6 (8.4)	621	678.9 (12.6)	708
GMVC				
Control	652.6 (14.4)	626	665.9 (11.5)	632
Low	651.6 (12.8)	633	685.5 (11.9)	680
Mid	675.3 (9.4)	633	680.9 (15.5)	660
High	674.1 (9.5)	645	697.3 (10.6)	715

### Body Weights and Clinical Signs

Growth curves are provided in Figure 2. Body weights of males in the mid- and high-level GVC exposure groups were below control values throughout the study (Figure 2a). Body weights of high-level GVC females were below control weights during most of the study and body weights of mid-level GVC females were below control values from d 122 to the end of the study at most time points. In the GMVC animals, body weights of high-level male and female rats remained below control values for most of the study (Figure 2b). Body weights of mid-level males and mid-level females were below control values for some or most of the study.

**Figure:**
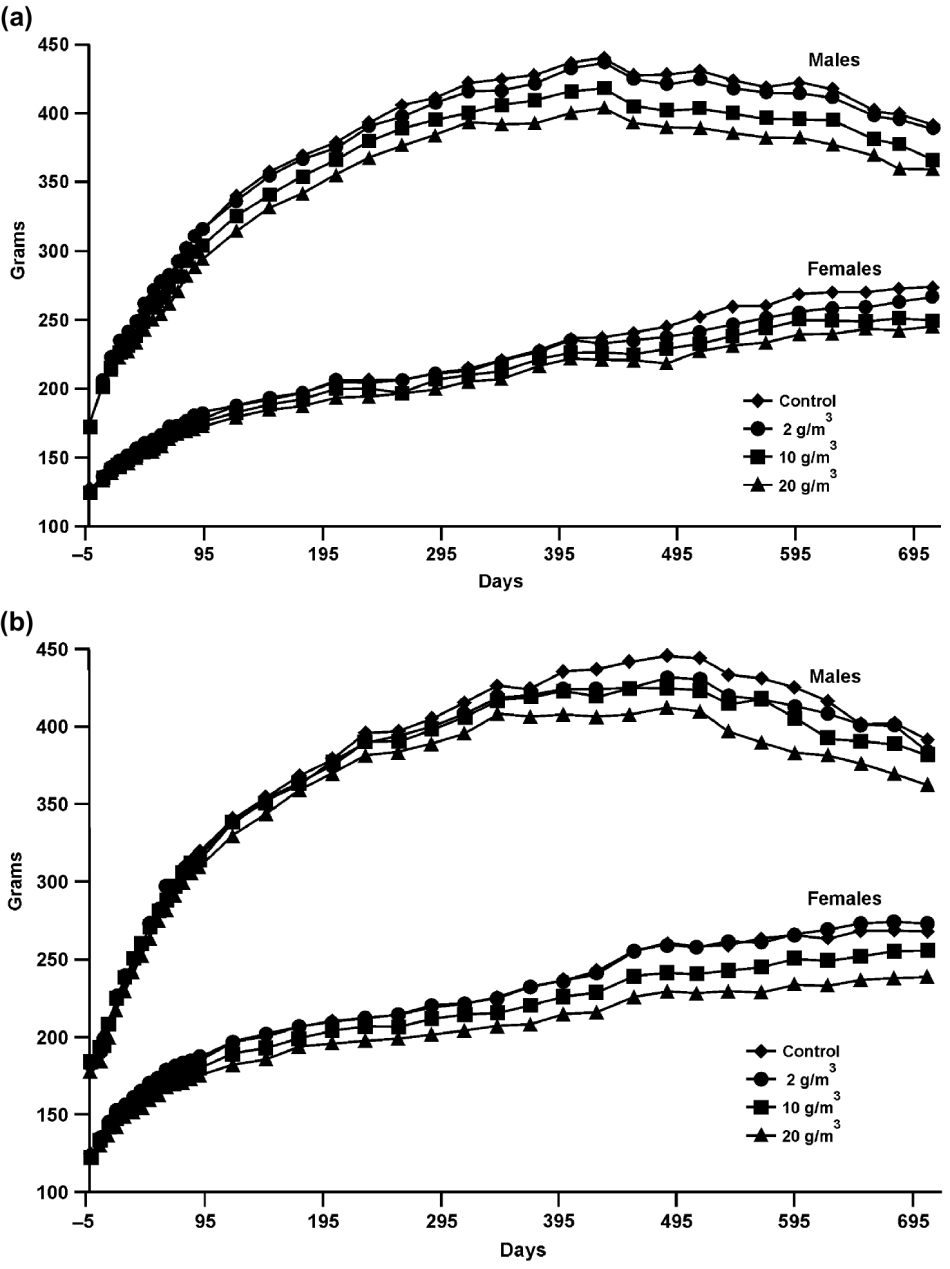
**FIGURE 2.** Growth curves: (a) Growth curves for male and female rats exposed to air or GVC. (b) Growth curves for male and female rats exposed to air or GMVC.

#### 

##### Clinical signs of toxicity

Rats exposed to GVC and GMVC displayed no apparent clinical signs of toxicity related to exposures. As this was a chronic toxicity evaluation, many of the observations during the second year were related to aging (e.g., mammary masses, jaundice).

##### Terminal body weights and organ weights at final sacrifice

Body weights at final sacrifice of high-level male and mid-and high-level female GVC-treated animals were significantly below control values (Table 4); weights of high-level males were 90.6% of controls and the mid- and high-level GVC females were 90.5% and 91.6% of controls, respectively. Male and female rats exposed to high-level GMVC had significantly reduced body weights at final sacrifice (Table 4); weights were 91.5% and 91.8% of control, respectively.

**TABLE 4.** Terminal Body Weight and Selected Organ Weights

	Terminal body weight, mean ± SD (g) (n)		Kidney weight, mean ± SD (g) (n)		Testes/ovaries weight, mean ± SD (g)		Lung weight, mean ± SD (g)	
Target (g/m^3^)	Male	Female	Male	Female	Male	Female	Male	Female
GVC								
0	377.5 ± 31.3 (18)	273.9 ± 20.5 (29)	3.284 ± 2.189 (18)	1.747 ± 0.104 (29)	4.889 ± 2.113 (18)	0.713 ± 3.246 (29)	2.191 ± 0.751 (18)	1.343 ± 0.125 (29)
2	388.3 ± 24.0 (22)	261.5 ± 24.1 (27)	2.761 ± 0.199 (22)	1.948 ± 0.780 (27)	6.133 ± 2.135 (21)	0.104 ± 0.018 (27)	1.958 ± 0.262 (22)	1.366 ± 0.203 (27)
10	357.8 ± 36.3 (26)	247.8 ± 22.4^*b*^ (27)	2.834 ± 0.534 (26)	1.853 ± 0.238^*a*^ (27)	5.649 ± 2.678 (25)	0.148 ± 0.158 (27)	2.115 ± 0.446 (26)	1.562 ± 0.740 (27)
20	341.9 ± 60.7^*a*^ (17)	251.0 ± 19.4^*b*^ (33)	2.857 ± 0.256 (17)	1.840 ± 0.122^*a*^ (33)	4.963 ± 2.626 (17)	0.323 ± 0.889 (33)	2.389 ± 0.674 (17)	1.351 ± 0.130 (32)
GMVC								
0	390.0 ± 18.2 (14)	265.4 ± 33.3 (22)	2.906 ± 0.230 (14)	1.951 ± 0.348 (22)	5.183 ± 3.133 (14)	0.117 ± 0.099 (22)	1.917± 0.281 (14)	1.562 ± 0.503 (22)
2	375.5 ± 35.2 (16)	273.5 ± 20.4 (30)	3.053 ± 0.598 (16)	1.900 ± 0.171 (30)	5.378 ± 1.948 (16)	0.126 ± 0.062 (30)	2.237 ± 0.566 (16)	1.366 ± 0.145 (30)
10	368.7 ± 28.5 (20)	256.3 ± 21.9 (30)	2.960 ± 0.236 (20)	1.908 ± 0.171 (30)	5.996 ± 2.419 (20)	0.141 ± 0.174 (30)	2.103 ± 0.411 (20)	1.360 ± 0.305 (30)
20	357.0 ± 28.4^*b*^ (19)	243.6 ± 15.7^*a*^ (33)	3.054 ± 0.365 (19)	1.933 ± 0.155 (33)	6.324 ± 2.448 (19)	0.105 ± 0.022 (33)	2.128 ± 0.570 (19)	1.374 ± 0.278 (33)
^*a*^Mean significantly different from control; data nonhomogeneous by Bartlett's test. Means compared using a modified *t*-test.								
^*b*^Mean significantly different from control; data homogeneous by Bartlett's test. Means compared using Dunnett's test of significance.								

Selected target organ weights are presented in Table 4. The absolute kidney weights for mid- and high-level GVC female rats were significantly greater than mean control values; however, weight did not correlate with kidney pathology findings. No significant effects on testes or ovary weights or lung weights were seen.

### Histopathology

#### 

##### Proliferative lesions

A summary of proliferative lesions is presented in Table 5. Proliferative lesions were more common among male than female rats exposed to gasoline vapor condensate with or without MTBE. Proliferative lesions common to males in both studies included renal-tubule adenomas and carcinomas, squamous-cell carcinoma of the nasal passages, testicular adenomas and mesothelioma, thyroid follicular-cell adenomas, and mononuclear-cell leukemia. Only in the case of renal tubule adenomas and carcinomas,- were statistically significant changes, attributable to gasoline vapor exposure, present in the same tissue in both studies. The only proliferative lesion common to females in both studies was mononuclear-cell leukemia. Fibroadenoma of the mammary gland was significantly increased among females exposed to GMVC.

**TABLE 5a.** GVC: Summary of Select Proliferative Lesions in Male and Female Rats

Tissue	Diagnosis	Control (0 g/m^3^)	Low (2 g/m^3^)	Mid (10 g/m^3^)	High (20 g/m^3^)
**Males**					
Kidney	*Number examined*	*50*	*50*	*50*	*50*
	Adenoma, renal tubule	1 (2%)	1 (2%)	4 (8%)	0 (0%)
	Carcinoma, renal tubule	0 (0%)	0 (0%)	3 (6%)	0 (0%)
	Renal tubule adenoma and carcinoma, combined	1 (2%)	1 (2%)	7 (14%)	0 (0%)
Nasal passages (Note that the same tumor may occur at more than one level.)	Turbinate level 2				
	*Number examined*	*50*	*50*	*50*	*50*
	Carcinoma, squamous cell	0 (0%)	0 (0%)	0 (0%)	1 (2%)
	Turbinate level 3				
	*Number examined*^*a*^	*50*	*50*	*49*	*50*
	Carcinoma, squamous cell^*b*^	0 (0%)	0 (0%)	0 (0%)	3 (6%)
	Turbinate level 4				
	*Number examined*	*50*	*50*	*49*	*50*
	Carcinoma, squamous cell	0 (0%)	1 (2%)	0 (0%)	3 (6%)
Testes	*Number examined*	*50*	*49*	*50*	*50*
	Mesothelioma, malignant^*c*^	0 (0%)	0 (0%)	4 (8%)	0 (0%)
	Adenoma, interstitial cell	48 (96%)	46 (94%)	50 (100%)	49 (98%)
Thyroid	*Number examined*	*50*	*29*	*27*	*50*
	Hyperplasia, follicular cell^*d,*^^*e*^	1 (2%)	0 (0%)	2 (7%)	6 (12%)
	Average severity	0.0 ± 0.1	0.0 ± 0.0	0.1 ± 0.4	0.2 ± 0.5
	Adenoma, follicular cell	0 (0%)	2 (7%)	0 (0%)	2 (4%)
	Carcinoma, follicular cell^*c*^	0 (0%)	0 (0%)	2 (7%)	0 (0%)
	Follicular cell adenoma and carcinoma, combined	0 (0%)	2 (7%)	2 (7%)	2 (4%)
Spleen	*Number examined*	*50*	*34*	*38*	*50*
	Leukemia, mononuclear cell	32 (64%)	23 (68%)	25 (66%)	32 (64%)
Tissue	Diagnosis	Control (0 g/m^3^)	Low (2 g/m^3^)	Mid (10 g/m^3^)	High (20 g/m^3^)
**Females**					
Spleen	*Number examined*	*50*	*25*	*32*	*50*
	Leukemia, mononuclear cell^*c*^	13 (26%)	14 (56%)	18 (56%)	15 (30%)
^*a*^Mid-dose turbinate levels 3 and 4 for one animal were autolytic, resulting in an *n* of 49.					
^*b*^Significant trend for increased incidence with increasing exposure concentration, Cochran–Armitage test.					
^*c*^Significant trend for increased incidence with increasing exposure concentration, Fisher's exact test.					
^*d*^Average ± SD of the severity score for all animals examined (both affected and unaffected). Unaffected animals were assigned a severity score of zero.					
^*e*^Significant increasing trend with exposure concentration with the Cochran–Armitage and logistic tests.					

##### Renal-tubule adenoma and carcinoma

In GVC-exposed male rats, the occurrence of renal-tubule adenoma and carcinoma peaked at the mid-dose level with incidences of 4/50 and 3/50, respectively. There were no renal-tubule adenomas or carcinomas in the high-level males. The incidences of adenomas or carcinomas alone were not statistically different from control values, but the combined adenoma and carcinoma incidences were statistically significant. Pairwise tests did not demonstrate any significant differences between groups in the combined incidence of renal adenoma + carcinoma. Similarly, male rats exposed to the mid-dose level of GMVC demonstrated significant increases in the incidence of renal-tubule adenomas (6/50; 12%) compared to controls (0/50). There was also a quantitative rise in renal-tubule carcinomas in the males (1/50; 2% in low-level males; and 1/50; 2% in high-level males). When incidences of adenomas and carcinomas were combined, there was a statistically significant trend toward an increased incidence, and the incidence of combined adenomas and carcinomas was statistically significant at the mid-dose group. The reason for the lack of renal neoplasms in the high-level group of male rats exposed to GVC is unknown.

##### Nasal squamous-cell carcinoma

In GVC-exposed animals, the incidence of squamous-cell carcinoma in the nasal passages (turbinate levels 2–4) was elevated in the high-level group of male rats, with tumor incidence of 1/50, 3/50, and 3/50 for levels 2, 3, and 4, respectively, in a total of 3 animals. In these three animals, the tumors were large and spanned over two or three turbinate levels. At turbinate level 3 there was a statistically significant trend for increased incidence with rising exposure concentration. In contrast to this, no significant change in squamous-cell carcinoma occurred in nasal sections at any turbinate level in male or female rats inhaling GMVC. The squamous-cell carcinomas in all of the male nasal specimens had morphologies consistent with origination from the oral mucosa and varying degrees of invasion into the nasal passages.

##### Testicular interstitial-cell adenomas

The background incidence of testicular interstitial cell adenomas in these studies was 96% in the GVC controls and 86% in GMVC controls. These incidences are not unexpected for aged rats. Exposure to GVC did not alter the incidence of this lesion. The incidence of interstitial-cell adenoma rose with increasing GMVC exposure concentration, but there were no significant group differences. The lower control incidence occurring in GMVC compared to GVC studies likely contributed to the statistical findings, because incidences in animals exposed to the two test materials were similar, ranging from 94 to 100%.

##### 

*Malignant mesothelioma* Malignant mesothelioma was also identified in testes of mid-dose level rats inhaling GVC and in all dose levels of rats inhaling GMVC. No malignant mesothelioma was found in the control animals of either study. Only in the GVC study did statistical analysis indicate a trend toward increasing incidence with exposure concentration.

##### Thyroid adenomas and carcinomas

Thyroid adenomas and carcinomas occurred in males exposed to GVC or GMVC, but only the incidence of follicular-cell carcinoma among GVC-exposed rats showed a significant trend with increasing exposure concentration. Again, as in the case of background incidences of testicular adenoma, control incidences of thyroid adenoma and carcinoma (zero in the GVC study) may have influenced the statistical outcome. However, the incidence (7%) in the mid-level group of GVC males is considerably higher than that reported in control F344 rats exposed to air (average incidence of 1% in 21 studies, range of 0 carcinomas in 52 rats to 1 in 45; NTP, 1999).

##### Mononuclear-cell leukemia (MCL)

Mononuclear-cell leukemia (MCL) is a well-documented finding in aged F344 rats. It occurred at a high incidence among control male and female rats as well as in rats inhaling GVC or GMVC. Results for spleen, the defining organ for this disease, are shown for males and females in Table 5. In GVC-exposed females and GMVC-exposed males, there was a statistically significantly elevated incidence of splenic MCL as a function of exposure concentration but there were no significant differences among exposure groups. The MCL is not considered to be treatment related, but rather attributable to sampling bias in the low- and mid-dose groups, where only spleens from animals showing gross lesions at necropsy were examined histologically.

##### Mammary-gland fibroadenoma

The incidence of mammary-gland fibroadenoma showed a trend for increased incidence with GMVC exposure, but there were no group differences. Interpretation of this finding is problematic due to the atypically low control incidence of 0% in control females. Control incidences for this neoplasm were reported to range from 27 to 40% (Boorman et al., 1990b). The control incidence in the parallel GVC study was 4/40 (8%). Additionally, increases in this neoplasm have not been observed in previous studies with neat MTBE or wholly vaporized gasoline (Cruzan et al., 2007; MacFarland et al., 1984). Therefore, it is judged unlikely that the increased incidence in female mammary-gland fibroadenoma was treatment-related.

### Nonproliferative Lesions

Summaries of nonproliferative lesions are presented in Tables 6, 7, and 8. Chronic progressive nephropathy was seen in the kidneys of both genders of all treatments groups. The incidence in GVC females was less than males, while in GMVC animals the incidences were similar in males and females. Other nonproliferative lesions included nasal respiratory and olfactory epithelial degeneration. The nasal lesions were more prevalent among animals inhaling GVC.[Table T0009]

**TABLE 5b.** GMVC: Incidences of Proliferative (Neoplastic and Hyperplastic) Lesions in Rats

		Control (0 g/m^3^)	Low (2g/m^3^)	Mid (10 g/m^3^)	High (20 g/m^3^)
**Males**					
Kidney	*Number examined*	*50*	*50*	*50*	*50*
	Adenoma, renal tubule	0 (0%)	0 (0%)	6 (12%)^*a*^	2 (4%)
	Carcinoma, renal tubule	0(0%)	1(2%)	0(0%)	1(2%)
	Renal tubule adenoma and carcinoma, combined^*b*^	0(0%)	1(2%)	6(12%)^*c*^	3(6%)
Nasal sections (Note that the same tumor may appear in more than one level.)	Turbinate level 1				
	* Number examined*	*50*	*50*	*50*	*50*
	* *Hyperplasia	3(6%)	0(0%)	3(6%)	4(8%)
	* *Average severity	0.2	0.0	0.1	0.2
	Turbinate level 2				
	* Number examined*	*50*	*50*	*50*	*50*
	* *Hyperplasia	0(0%)	2(4%)	2(4%)	3(6%)
	* *Average severity	0.0	0.1	0.1	0.2
	* *Carcinoma, squamous cell	1(2%)	0(0%)	0(0%)	0(0%)
	Turbinate level 3				
	* Number examined*	*50*	*50*	*50*	*50*
	* *Carcinoma, squamous cell	0(0%)	1(2%)	0(0%)	1(2%)
	Turbinate level 4				
	* Number examined*	*50*	*50*	*50*	*50*
	* *Carcinoma, squamous cell	0(0%)	2(4%)	0(0%)	2(4%)
Thyroid	*Number examined*	*50*	*35*	*31*	*50*
	Hyperplasia, follicular cell	0(0%)	0(0%)	1(3%)	0(0%)
	Average severity	0.0 ± 0.0	0.0 ± 0.0	0.1 ± 0.5	0.0 ± 0.0
	Adenoma, follicular cell	2(4%)	0(0%)	0(0%)	3(6%)
	Carcinoma, follicular cell	1(2%)	0(0%)	1(3%)	3(6%)
	Follicular cell adenoma and carcinoma, combined	3(6%)	0(0%)	1(3%)	5(10%)
Testes	*Number. examined*	*50*	*50*	*50*	*50*
	Adenoma, interstitial cell^*d*^	43(86%)	47(94%)	48(96%)	50(100%)^*e*^
	Mesothelioma	0 (0%)	1(2%)	1(2%)	2(4%)
Spleen	*Number examined*	*50*	*39*	*38*	*50*
	Leukemia, mononuclear^*f*^	27(54%)	31(79%)^*e*^	31(82%)^*e*^	31(62%)
**Females**					
Spleen	*Number examined*	*50*	*22*	*24*	*50*
	Leukemia, mononuclear	27(54%)	12(55%)	11(46%)	23(46%)
Mammary Gland	*Number examined*	*49*	*20*	*22*	*47*
	Fibroadenoma^*f*^	0(0%)	3(15%)^*e*^	2(9%)	5(11%)^*e*^
^*a*^Incidence is significantly different from controls and from the 2-g/m^3^ group, Fisher's exact test.					
^*b*^A positive trend toward increased incidence with increasing exposure concentration found with the Cochran–Armitage test.					
^*c*^ Incidence is significantly different from controls, Fisher's exact test.					
^*d*^A positive trend with increasing exposure concentration found with the Fisher's exact, Cochran–Armitage and Logistic tests.					
^*e*^Incidence significantly different from controls, Fisher's exact test.					
^*f*^A positive trend with increasing exposure concentration found with the Fisher's exact test.

**TABLE 6.** Comparison of Chronic Progressive Nephropathy in Rats Inhaling GMVC and GVC for 2 Years

	0 g/m^3^	2 g/m^3^	10 g/m^3^	20 g/m^3^
**GVC**				
Males				
Incidence^*a*^	49/50 (98%)	49/50 (98%)	50/50 (100%)	50/50 (100%)
Average severity^*b*^	2.4 ± 0.85	2.5 ± 0.81	2.9 ± 0.79^*c*^	2.9 ± 0.77^*c*^
Females				
Incidence	27/50 (54%)	12/24 (50%)	16/25 (64%)	32/50 (64%)
Average severity	0.66 ± 0.69	1.0 ± 1.2	0.92 ± 0.86	0.86 ± 0.78
**GMVC**				
Males				
Incidence	44/50 (88%)	47/50 (94%)	50/50 (100%)	46/50 (92%)
Average severity	1.9 ± 0.99	2.1 ± 0.92	2.6 ± 0.64^*c*^	2.7 ± 0.94^*c*^
Females				
Incidence	42/50 (84%)	17/20 (85%)	15/21 (71%)	43/50 (86%)
Average severity	1.0 ± 0.64	1.0 ± 0.56	1.0 ± 0.80	1.5 ± 0.91^*c*^
^*a*^Incidence (percent).				
^*b*^Average ± SD of severity score of the number of animals examined (both affected and unaffected). Unaffected animals were assigned a severity score of zero.				
^*c*^Significantly different from controls (*p* < .05).

**TABLE 7.** Incidence and Severity of Respiratory Epithelial Degeneration

	GVC				GMVC			
	0 g/m^3^	2 g/m^3^	10 g/m^3^	20 g/m^3^	0 g/m^3^	2 g/m^3^	10 g/m^3^	20 g/m^3^
**Males**								
Turbinate level 2								
Number examined	50	50	50	50	50	50	50	50
Number with lesions (%)	2 (4)	3 (6)	7 (14)	11 (22)^*a*^	0	1 (2)	2 (4)	3 (6)
Average severity^*b*^	0.0 ± 0.2	0.1 ± 0.2	0.2 ± 0.6	0.2 ± 0.5	–	0.0 ± 0.1	0.1 ± 0.4	0.1 ± 0.6
Turbinate level 3								
Number examined	50	50	49	50	50	50	50	50
Number with lesions (%)	1 (2)	0 (0)	7 (14)^*c*^	2 (4)	0 (0)	0 (0)	0 (0)	0 (0)
Average severity	0.0 ± 0.3	–	0.2 ± 0.5	0.0 ± 0.2	–	–	–	–
**Females**								
Turbinate level 2								
Number examined	49	49	50	50	50	50	50	50
Number with lesions (%)	8 (16)	11 (22)	18 (36)	26 (52)^*a,*^^*d,*^^*e*^	0 (0)	1 (2)	7 (14)	6 (12)^*a,*^^*f*^
Average severity	0.2 ± 0.5	0.3 ± 0.6	0.5 ± 0.8	0.8 ± 0.8^*c*^	–	0.0 ± 0.1	0.3 ± 0.7	0.2 ± 0.6
Turbinate level 3								
Number examined	49	49	50	50	50	50	50	50
Number with lesions (%)	2 (4)	0 (0)	8 (16)^*g*^	12 (24)^*a,*^^*e*^	0 (0)	0 (0)	0 (0)	0 (0)
Average severity	0.0 ± 0.2	–	0.2 ± 0.5	0.4 ± 0.7	–	–	–	–
^*a*^Significant trend for increased incidence with increasing exposure level, Cochran–Armitage and logistic tests.								
^*b*^Average ± SD of severity score of the number of animals examined (both affected and unaffected). Unaffected animals were assigned a severity score of zero.								
^*c*^Significantly different from the low-level group, Fisher's exact test.								
^*d*^Overall severity/incidence is significantly greater than control, Kolmorogov–Smirnoff test.								
^*e*^Significantly different from the control and low-level groups, Fisher's exact test.								
^*f*^Significant trend for increased incidence with increasing exposure level, Fisher's exact test.								
^*g*^Significantly different from the low-level group, Fisher's exact test.

**TABLE 8. T0009:** Incidence and Severity of Olfactory Epithelial Degeneration

	GVC				GMVC			
	0 g/m^3^	2 g/m^3^	10 g/m^3^	20 g/m^3^	0 g/m^3^	2 g/m^3^	10 g/m^3^	20 g/m^3^
**Males**								
Turbinate level 2								
Number examined	50	50	50	50	50	50	50	50
Number with lesions (%)	0 (0)	0 (0)	2 (4)	2 (4)	0 (0)	5 (10)	7 (14)	6 (12)^*a*^
Average severity^*b*^	–	–	0.0 ± 0.2	0.0 ± 0.2	–	0.2 ± 0.5	0.2 ± 0.6	0.2 ± 0.7
Turbinate level 3								
Number examined	50	50	49	50	50	50	50	50
Number with lesions (%)	15 (30)	3 (6)	7 (14)^*c*^	4 (8)^*a*^	2 (4)	1 (2)	7 (14)	7 (14)^*d*^
Average severity	0.3 ± 0.5	0.1 ± 0.4	0.2 ± 0.4	0.1 ± 0.3	0.0 ± 0.2	0.0 ± 0.3	0.2 ± 0.6	0.3 ± 0.8
Turbinate level 4								
Number examined	50	50	49	50	50	50	50	50
Number with Lesions (%)	18 (36)	10 (20)	6 (12)	1 (2)^*e,*^^*f,*^^*g*^	0	0	1 (2)	1 (2)
Average Severity	0.4 ± 0.5	0.2 ± 0.4	0.2 ± 0.6	0.0 ± 0.1	–	–	0.0 ± 0.1	0.0 ± 0.1
**Females**								
Turbinate level 2								
Number examined	49	49	50	50	50	50	50	50
Number with Lesions (%)	0 (0)	0 (0)	7 (14)	2 (4)	4 (8)	5 (10)	6 (12)	10 (20)
Average severity	–	–	0.3 ± 0.7	0.0 ± 0.2	0.1 ± 0.4	0.2 ± 0.5	0.2 ± 0.6	0.3 ± 0.6
Turbinate level 3								
Number examined	49	49	50	50	50	50	50	50
Number with lesions (%)	15 (31)	13 (27)	11 (22)	13 (26)	4 (8)	2 (4)	10 (20)	8 (16)
Average severity	0.3 ± 0.5	0.3 ± 0.5	0.3 ± 0.5	0.3 ± 0.5	0.1 ± 0.5	0.1 ± 0.3	0.4 ± 0.9	0.3 ± 0.8
Turbinate level 4								
Number examined	49	49	50	49	50	50	50	50
Number with lesions (%)	21 (43)	10 (20)	7 (14)^*g*^	2 (4)^*e,*^^*f,*^^*g*^	3 (6)	0 (0)	0 (0)	3 (6)
Average severity	0.4 ± 0.5	0.2 ± 0.4	0.2 ± 0.4	0.0 ± 0.2	0.0 ± 0.3	–	–	0.1 ± 0.2
^*a*^Significant trend for increasing incidence with exposure concentration, Cochran–Armitage, logistic, and Fisher's exact tests.								
^*b*^Average ± SD of severity score of the number of animals examined (both affected and unaffected). Unaffected animals were assigned a severity score of zero.								
^*c*^Significantly greater than the low-level group, Fisher's exact test.								
^*d*^Significant trend for increased incidence with increasing exposure level, Cochran–Armitage and logistic tests.								
^*e*^Significant trend for decreased incidence with increasing exposure level, Cochran–Armitage, logistic, and Fisher's exact tests.								
^*f*^Overall severity/incidence is significantly less than control, Kolmorogov–Smirnoff test.								
^*g*^Significantly less than control, Fisher's exact test.								

#### 

##### Chronic progressive nephropathy

Kidneys of nearly all male control and GVC-exposed rats displayed evidence of chronic progressive nephropathy (Table 6), with no significant difference in the incidence among the groups. However, overall combination of incidence and severity of the lesion, assessed using the Kolmogorov–Smirnoff test, was significantly greater in the mid- and high-level exposure groups. This finding in males is consistent with male-rat-specific light hydrocarbon-induced alpha-2μ globulin overload nephropathy that was reported previously in studies with wholly vaporized unleaded gasoline (MacFarland et al., 1984). In F344 male rats, after approximately 1 yr of age, alpha-2μ globulin overload nephropathy is essentially masked by age-related chronic progressive nephropathy, since both forms of nephropathy share similar if not identical diagnostic hallmarks. Therefore the effects of alpha-2μ globulin overload nephropathy typically exacerbate the severity of chronic progressive nephropathy, rather than incidence, since essentially all males surviving past 1 yr of age will develop chronic progressive nephropathy. Thus, the statistically significant worsening of chronic progressive nephropathy in male rats with higher levels of exposure is consistent with an effect induced by the test substance.

Among females in the GVC study chronic progressive nephropathy occurred in all groups, including controls, but the female incidence was approximately 60% of that seen among males. The combined incidence and severity of the lesion in the GVC-exposed females (as reflected in the severity score and Kolmogorov–Smirnoff assessment) were not significantly different from that of the control females.

The incidence of chronic progressive nephropathy among control male (88%) and that among control female (84%) rats in the GMVC study were similar. The incidences among male and female rats inhaling GMVC were not significantly changed over the incidence in the respective controls. However, the combined incidence and severity of this lesion was significantly elevated in mid- and high-level males and in high-level females, compared to the corresponding control values. The reason for the higher incidence of chronic progressive nephropathy in females, especially controls in the GMVC study compared to the GVC study, is not known.

##### Nasal respiratory epithelial degeneration

In the GVC study, degeneration of the nasal epithelium, characterized primarily by accumulation of globular, homogeneous, brightly eosinophilic material in the cytoplasm of respiratory and/or olfactory epithelial cells, was present at minimal to moderate severity in all groups of animals (Table 7). The finding was somewhat sporadic, but its incidence in the respiratory epithelium of the nasal passages of male rats at turbinate level 2 (approximately at the level of the incisive papilla) showed a significant trend for increased incidence with rising level of exposure. The combination of incidence and severity of the lesion at turbinate level 2, however, is not significantly different among the male dose groups. There was also a significant trend for increased incidence of the degenerative change in respiratory epithelium at turbinate level 3 (second palatal ridge) in male rats. The incidence of the lesion in the mid-level group was significantly greater than in the low-level group.

In the GVC study, the incidence of nasal respiratory epithelial degeneration for female rats including control was somewhat greater than that of the males. Significant trends for increases in incidence or severity with increasing exposure level of the respiratory epithelial lesion were noted at both turbinate levels 2 and 3 of the females, with incidences at levels 2 and 3 in the high-level group significantly greater than the control and low-level groups. The incidence at turbinate level 3 in the mid-level group was significantly higher than in the low-level group. The combination of incidence and severity of the respiratory epithelial lesion was significantly greater only in the female high-level GVC exposure group and only at turbinate level 2. The lesion in both genders is likely induced by exposure to the test substance.

The incidences of respiratory epithelial degeneration in the GMVC study were less than those in the GVC study. Among females, there was a statistically significant increase in epithelial degeneration at turbinate level 2, but there were no group differences. No lesions were observed in GMVC males or females at turbinate level 3.

##### Olfactory epithelial degeneration

In the GVC study there was a relatively high background incidence of olfactory epithelial degeneration at turbinate levels 3 and 4 among control males and females (Table 8). Except for control females at turbinate level 2, the incidences in controls in the GVC were notably greater than seen in the GMVC study. In males, GVC exposure produced a statistically significant exposure-related decrease in the degeneration of olfactory epithelium in turbinate levels 3 and 4 (level of the first molar) in the nasal passages (Table 8). At turbinate level 4, the incidence in the high-level male group was significantly less than in the control and low-level groups. This exposure-response relationship was also present in the females at turbinate level 4, where the incidence in the mid- and high-level rats was less than in controls.

In males, GMVC exposure significantly increased the incidence of olfactory epithelial degeneration at turbinate level 2, but there were no associated differences among exposure groups. The incidence of degenerative lesions at turbinate level 4 was low and nonsignificant. Among females, the only background incidence of olfactory epithelial degeneration occurred at turbinate level 2. There was no significant altered incidence among the GMVC-exposed female animals. Minimal incidence of olfactory epithelial degeneration was seen at turbinates levels 3 and 4.

## DISCUSSION

These chronic inhalation studies were conducted to compare the toxicity and potential carcinogenicity of evaporative emissions from unleaded gasoline and gasoline containing the oxygenate, methyl tertiary-butyl ether (MTBE), and to comply with fuel and fuel additive registration requirements under Section 211(b) of the Clean Air Act. In previous chronic inhalation studies of neat MTBE, F344 rats were exposed to 0, 400 (∼1.4 g/m^3^), 3000 (∼10.8 g/m^3^), and 8000 (∼28.8 g/m^3^) ppm (Bird et al., 1997; Mennear, 1997). An increased incidence of renal tubular adenomas and/or carcinomas was observed in males exposed to 8000 ppm (28.8 g/m^3^), and an elevated incidence in testicular tumors was found among rats exposed to 3000 or 8000 ppm (10.8–28.8 g/m^3^). In comparison, the concentrations of MTBE in the evaporative emissions tested in these studies were approximately 20% of the total vapor concentration, or approximately 0.4, 2, and 4 g/m^3^ in the low, mid, and high GMVC groups, respectively.

The effects of treatment with GVC and GMVC on survival (none), clinical observations (none), body weights (decreases), and organ weights (few) were comparable between the two studies. Therefore, the remainder of the discussion focuses on comparisons of proliferative and nonproliferative lesions between GVC- and GMVC-exposed rats.

The major proliferative lesions, attributable to vapor condensate inhalation, with or without MTBE, were renal-tubule adenomas and carcinomas in males. The occurrence of combined renal adenomas and carcinomas peaked in the mid-exposure-level groups for animals exposed to GVC (7/50 = 14%) and GMVC (6/50 = 12%). The occurrence of renal tumors in males is consistent with results of previous inhalation studies on wholly vaporized unleaded gasoline (MacFarland et al., 1984) and neat MTBE (Bird et al., 1997). The presence of MTBE in the vapor condensate did not enhance tumor incidence over that seen with GVC. The lack of additivity of renal tubule tumor incidence in GMVC- versus GVC-exposed rats may be explained by the findings of Bird et al. (1997) showing no renal tubule tumors among rats inhaling 1.4 g MTBE/m^3^, a concentration bracketed by those in this study (0.4–4 g/m^3^). Bird et al. (1997) reported significant increases in renal tumors only in animals inhaling ≥10.8 g MTBE/m^3^.

The development of renal tumors in the male rats in this study inhaling a complex mixture of relatively low-molecular-weight hydrocarbons is believed to be mediated by increased alpha-2μ globulin accumulation and subsequent increased renal-tubule cell turnover. However, this process is masked in older rats through the development of chronic progressive nephropathy, so a direct correlation between alpha-2μ globulin-induced nephropathy, as might be assessed in a 90-d study, and tumor incidence in these 2-yr studies cannot be made. The renal tumor incidence in GVC- and GMVC-exposed male rats did not directly correlate with the combined incidence and severity of chronic nephropathy. This finding is somewhat contrary to a report of Doi et al. (2007), where analysis of results of 2-yr National Toxicology Program (NTP) bioassays indicated that the severity of chronic nephropathy best correlated with renal tumor responses. Since renal tumors development secondary to alpha-2μ accumulation does not occur in humans, the relevance of the vapor condensate-induced renal tumors in this study is not predictive of human health hazard (Baetcke et al., 1991; Hard, 1998; Hard and Khan, 2004; U.S. EPA, 1991).

The background incidence of testicular tumors in rats in these studies was high, but not inconsistent with reports of Boorman et al. (1990c) and Gopinath et al. (1987). Background incidences were higher than those reported for control males in NTP inhalation studies where the range was 46–90% for animals fed NIH-07 diet and 66–84% for rats fed NTP-2000 diet. There was no evidence that GVC enhanced the testicular adenoma incidence, but there was a trend for increased incidence with GMVC exposure concentration. However, the incidences among rats inhaling GMVC and GVC at any level are comparable, suggesting the trend for increased incidence among GMVC males may have been driven by the lower incidence seen in concurrent controls in that study. Other MTBE toxicity studies documented increases in testicular interstitial cell adenomas (Bird et al., 1997; Belpoggi et al., 1995). However, in a review of the mechanisms of MTBE-induced carcinogenicity, the overall evidence for MTBE induction of testicular cell adenoma was considered to be equivocal (Cruzan et al., 2007). Further, the human health relevance of interstitial cell tumors in rats has been called into question, suggesting little predictive value for humans (Prentice & Meikle, 1995; Cook et al., 1999).

Testicular mesothelomia was a significant finding in GVC but not GMVC rats. The incidence of testicular mesothelioma in the mid-level GVC group (4/50; 8%) is approximately threefold greater than the average incidence in male control F344 rats exposed to air in inhalation toxicology studies (28 tumors in 1055 rats or 2.7%; NTP, 1999) and may be regarded as an effect of exposure to the test substance. Most mesotheliomas are thought to originate in the tunica vaginalis of the scrotum (Hall, 1990), and those found in this study are consistent with an origination from this investment of the testes. As with this study, there were no mesotheliomas found in the testes or other tissues of the control male animals of the GMVC study, although there was a quantitative rise in incidence (2 in 50 animals or 4%) of the tumor in the testes of the high-level GMVC-exposed rats and sporadic incidence of the tumor in other tissues of GMVC-exposed male rats. The absence of testicular mesothelioma in control animals, where 1 or 2 tumors might normally be expected to be found in 50 animals (NTP, 1999), may have influenced the statistical analysis to indicate a significant exposure-related incidence in the mid-level GVC-exposed male rats when no causal increase is actually present.

Among GVC-exposed male rats, there was a trend for increased thyroid carcinomas. There was no statistical evidence for elevated incidence of adenomas or of adenomas and carcinomas combined; however, the incidence of thyroid follicular carcinomas and adenomas combined is higher than what might be expected (NTP, 1999). Further, follicular epithelial hyperplasia at minimal to mild severity was noted in male rats of the control, mid-, and high-level groups (2–12% incidence) and also displayed an increased incidence with increasing exposure level. There were no significant changes in thyroid follicular-cell adenomas, carcinomas, or combined adenomas and carcinomas or thyroid follicular epithelial hyperplasia in males inhaling GMVC. This is possibly due to the higher incidence of adenomas (4%) and carcinomas (2%) in the controls in the GMVC study. An increased incidence of thyroid tumors was not reported among rats inhaling up to 8000 ppm MTBE (Bird et al., 1997). Taken together, these results suggest that thyroid follicular proliferative lesions in the male animals were produced by GVC exposure.

Despite the proliferative lesions in the thyroid glands of male rats inhaling GVC, the relevance of the test article exposure to human risk might be questioned, given that mechanisms of chemical thyroid carcinogenesis are believed to be different between humans and rodents (U.S. EPA, 1998). Mutation of thyroid follicular cell DNA may lead directly to cancer and is the only mechanism verified to be carcinogenic in humans, but rodents are believed to be more susceptible to carcinogenic processes involving stimulation of thyroid follicular cell growth through disruption of pituitary–thyroid hormonal physiology. Mutagenesis of thyroid follicular cells and hormone disruption were not evaluated in this study.

Nasal squamous-cell carcinomas seen in this study originated in the oral mucosa. Spontaneous oral squamous cell carcinoma incidence in F344 rats is rare (Haseman et al., 1990; NTP, 1999). While there was a significant trend toward an increase in incidence of nasal squamous cell carcinomas in male rats inhaling GVC, there were no significant differences among dose groups. Further, this tumor type was identified in the control female group in the GVC study and among males in the GMVC study without evidence of statistical significance. Therefore, it is difficult to attribute this lesion to gasoline vapor exposure, and there is no evidence that the lesion is enhanced by exposure to MTBE.

Degeneration of the respiratory and olfactory epithelium is a common, nonspecific finding in aged rats (Boorman et al., 1990d; Gopinath et al., 1987). The background incidences of respiratory and olfactory epithelial degeneration are notably higher in the GVC compared to the GMVC study. The reason for this is not totally clear, as rats were from the same source and housed identically. Further, the reasons for the differential effects of GVC (increased respiratory degeneration and decreased olfactory degeneration with increasing exposure level) and GMVC (increased incidence of olfactory lesions in turbinates 2 and 3 among males) are not obvious. Responses appear to be turbinate, gender, and exposure dependent.

In summary, a side-by-side comparison was conducted of the toxicity and carcinogenicity of gasoline vapor condensate and gasoline vapor condensate containing approximately 20% MTBE. The inhalation exposure systems were identical. Animals were obtained from the same source, were the same age, and were housed under identical conditions. The health effects resulting in F344 rats following 2 yr of GMVC or GVC inhalation were unequivocally comparable in the production of renal adenomas and carcinomas in male rats. Methyl tertiary-butyl ether was not identified as enhancing the production of these renal adenomas or adenomas and carcinomas compared to those induced by gasoline vapor condensate alone.
